# The Relationship between Burden and Financial Factors for Help-Seeking Older Adult Caregivers

**DOI:** 10.1093/geroni/igab046.2973

**Published:** 2021-12-17

**Authors:** Sara Qualls, Stacy Yun, Kendall Weber, Geffen Ferszt

**Affiliations:** 1 University of Colorado Colorado Springs, University of Colorado at Colorado Springs, Colorado, United States; 2 University of Colorado Colorado Springs, Colorado Springs, Colorado, United States; 3 University of Colorado at Colorado Springs, University of Colorado at Colorado Springs, Colorado, United States

## Abstract

Older adult caregivers often experience unique financial challenges (Schultz & Eden, 2016). The purpose of the present study was to explore the relationship between caregiver finances and caregiver burden for this sample. A sample of 131 caregivers seeking counseling services completed questionnaires assessing demographics, care recipient functioning (ADLs/IADLs), and finances (yearly income, impact of caregiving on finances). A majority, 74.8% of caregivers, indicated financial burden since caregiving. A hierarchical multiple regression was computed to predict caregiver burden. The caregiving characteristics block explained 8.1% of the total variance in caregiver burden, F(7, 123) = 1.54, n.s. Specifically, being younger was significantly associated with more caregiver burden. Adding the care recipient functioning block explained an additional 6.9% of the variance in caregiver burden, Fchange(2, 121) = 4.93, p < .01. The caregiving characteristics and care recipient functioning model accounted for 15% of the total variance in caregiver burden, F(9, 121) = 1.55, p < .05. Again, younger age uniquely predicted greater caregiver burden. Lastly, caregiving finance factors contributed an additional 13.5% of the variance, Fchange(4, 117) = 5.54, p < .001. Thus, the final caregiver characteristics, care recipient functioning, and caregiving finances model accounted for 28.5% of the total variance in caregiving burden, F(13, 117) = 3.59, p < .001. Specifically, having less income and greater monthly expenses related to caregiving predicted higher levels of caregiver burden. These findings imply that those with fewer resources may benefit from intervention for the heavier burden they perceive compared to peers with more financial resources.

